# ETS Proto-Oncogene 1 Suppresses MicroRNA-128 Transcription to Promote Osteogenic Differentiation Through the HOXA13/β-Catenin Axis

**DOI:** 10.3389/fphys.2021.626248

**Published:** 2021-03-03

**Authors:** Renyao Li, Ying Dong, Feipeng Li

**Affiliations:** ^1^School of Biological Science and Medical Engineering, Beihang University, Beijing, China; ^2^Naton Biotech (Beijing) Co., Ltd., Beijing, China

**Keywords:** E26 transformation-specific-1, microRNA-128, HOXA13, β-catenin, osteoporosis, osteogenic differentiation

## Abstract

ETS proto-oncogene 1 (ETS1) has been implicated in osteoporosis (OP), but the exact molecular mechanisms are complex. This work focuses on the impact of ETS1 on the osteogenic differentiation and the molecules involved. A mouse pre-osteoblast cell line MC3T3-E1 was used for *in vitro* experiments. ETS1 was upregulated during the process of osteogenic differentiation of MC3T3-E1 cells. Overexpression of ETS1 promoted expression of osteogenic markers, alkaline phosphate concentration, and calcareous accumulation in cells. ETS1 was found to specifically bind to miR-128 promoter to suppress its transcription, while miR-128 could target homeobox A13 (HOXA13). Therefore, ETS1 suppressed miR-128 transcription to upregulate HOXA13 expression. Overexpression of HOXA13 promoted the osteogenic differentiation ability of cells and increased the protein level of β-catenin. Either overexpression of miR-128 or downregulation of β-catenin by CWP232228, a β-catenin-specific antagonist, blocked the promoting roles of ETS1 in cells. To conclude, this study provided evidence that ETS1 suppresses miR-128 transcription to activate the following HOXA13/β-catenin axis, therefore promoting osteogenic differentiation ability of MC3T3-E1 cells. This finding may offer novel ideas for OP treatment.

## Introduction

Osteoporosis (OP) is the most frequent skeletal disease characterized by low bone mass and microarchitectural deterioration of bone tissue resulted from an imbalance in bone turnover, namely a greater rate of bone resorption by the osteoclasts than the rate of bone formation by the osteoblasts, leading to enhanced bone fragility and a consequent increase in fracture risk (Nitta et al., 2017; Anastasilakis et al., 2018; Briot et al., 2018). Postmenopausal women are the most vulnerable class to OP, especially those aging ones (Anastasilakis et al., 2018). The aim of the current pharmacological interventions is to correct the imbalance between bone resorption and bone formation which constitutes the pathophysiological foundation of this disease; however, the efficacy of the current treatments is below expectations since they can only modestly reduce the risk of non-vertebral fractures but cannot provide alternations for the lost bone (Appelman-Dijkstra and Papapoulos, 2015). Exploring novel therapeutic options for bone formation and OP control is of great importance, which requires more understanding in the molecules implicated in the pathogenesis of this disease.

ETS proto-oncogene 1 (ETS1) is a member of the ETS family that participates in the regulation of a wide array of cellular behaviors including cell proliferation, differentiation, apoptosis, angiogenesis, and so forth (Itoh et al., 2010). Aberrant expression of ETS1 was frequently observed in the progression of human malignancies and it mainly triggers tumor extracellular matrix degradation and metastasis (Wei et al., 2012; Xu et al., 2017; Yalim-Camci et al., 2019; Luo et al., 2020). Importantly, ETS1 has been noted to exert key functions in osteogenesis (Raouf and Seth, 2000). But the exact function of ETS1 in osteogenic differentiation and the implicated molecules are too complex to be fully explained. As a transcription factor, ETS1 can mediate the activity of downstream transcripts such as microRNAs (miRNAs) to exert its functions (Kern et al., 2012; Taylor et al., 2016). miRNAs are the mostly studied non-coding RNAs, and thanks to their functions in posttranscriptional regulation of mRNA expression, they are involved in many cellular and molecular activities and play important roles in many pathological processes including bone remodeling (Sun et al., 2016). Here, our integrated bioinformatic analysis precited that ETS1 possibly bind to the promoter region of miR-128. Importantly, miR-128 has been documented to suppress osteogenic differentiation during OP progression (Zhao et al., 2019). Intriguingly, homeobox A13 (HOXA13) was predicted as a target mRNA of miR-128 according to the bioinformatic analyses as well. A homologous gene of HOXA13, HOXA10, was reported to promote the osteogenic differentiation of bone marrow stem cells (Liao et al., 2013). HOXA13 has been suggested as a positive regulator of β-catenin (Qu et al., 2017), while the β-catenin activation is crucial for the process of osteoblast differentiation (Rossini et al., 2013). We speculated that HOXA13 possibly exerts a similar pro-osteogenic function. Taken together, our study hypothesized that ETS1 regulates miR-128 transcription to mediate HOXA13 expression, thereby promoting osteogenic differentiation and bone formation. Altered expression of these molecules was introduced in a mouse pre-osteoblast cell line MC3T3-E1 to validate the hypothesis.

## Materials and Methods

### *In vitro* Cell Culture, Transfection, and Differentiation

A mouse pre-osteoblast cell line MC3T3-E1 (Subclone 14, CRL-2593) was acquired from ATCC (Manassas, VA, United States). The cells were cultured in DMEM containing 10% FBS and 1% penicillin/streptomycin (all from Gibco, Carlsbad, CA, United States) at constant 37°C with 5% CO_2_ and 95% humidity. The medium was refreshed every 2 days.

The miR-128 mimic, ETS1 overexpressing vector (oe-ETS1), oe-HOXA13 and the corresponding negative control (NC) mimic as well as oe-NC for transfection were acquired from GenePharma Co., Ltd. (Shanghai, China). All transfection was performed using the Lipofectamine^TM^ 2000 (Invitrogen, Thermo Scientific Pierce, Rockford, IL, United States) according to the kit’s instructions. The transfection efficacy was determined 48 h later, and cells with stable transfection were harvested for the subsequent experiments. A Wnt/β-catenin-specific antagonist, CWP232228, was purchased from MedChemExpress (Monmouth Junction, NJ, United States) and used to treat the cells at a dose of 0.5 μmol.

The osteogenic differentiation of MC3T3-E1 cells was induced by the addition of 100 nM dexamethasone, 10 mM β-glycerol phosphate and 50 μg/mL ascorbic acid (all from Sigma-Aldrich Chemical Company, St. Louis, MO, United States) to the medium.

### Reverse Transcription Quantitative Polymerase Chain Reaction (RT-qPCR)

The TRIzol reagent (Invitrogen) was used to extract total RNA from cells. The RNA was transcribed to cDNA utilizing a Reverse Transcription Kit (Takara Biotechnology Ltd., Dalian, China). Next, a real-time qPCR was performed on a Bio-Rad CFX96 System using a SYBR Green Real-Time PCR Kit (Takara). The primer sequences were obtained from OriGene Technologies (Beijing, China) and listed in Table 1, where GAPDH and U6 were used as the internal controls for mRNAs and miRNA, respectively. Relative gene fold changes were measured using the 2^–ΔΔ*Ct*^ method.

**TABLE 1:** Primer sequences for RT-qPCR.

**Gene**	**Primer sequence (5′–3′)**
ETS1	F: CCAGAATCCTGTTACACCTCGG
	R: CAGCGTCTGATAGGACTCTGTG
miR-128	F: TCACAGTGAACCGGTCTC
	R: GAACATGTCTGCGTATCTC
HOXA13	F: CCCAAAGAGCAGACGCAGCCT
	R: GTGTAAGGCACGCGCTTCTTTC
RUNX2	F: CCTGAACTCTGCACCAAGTCCT
	R: TCATCTGGCTCAGATAGGAGGG
OCN (BGLAP)	F: GCAATAAGGTAGTGAACAGACTCC
	R: CCATAGATGCGTTTGTAGGCGG
OSX (SP7)	F: GGCTTTTCTGCGGCAAGAGGTT
	R: CGCTGATGTTTGCTCAAGTGGTC
miR-125a	F: CGGTCCCTGAGACCCTTTAAC
	R: GTGCAGGGTCCGAGGT
miR-21	F: ACACTCCAGCTGGGTAGCTTATCAGACTGA
	R: TGGTGTCGTGGAGTCG
NF1	F: CGGCTGCTTTGGAACAATCAGG
	R: GTGTATCTGCCACAGGCTTGTG
RET	F: TCAGTACACGGTGGTAGCCACT
	R: CGCCTCTTGTTTACTGCACAGG
SOX7	F: TGAATGCCTTCATGGTGTGGGC
	R: ACAGTGTCAGCGCCTTCCATGA
ING5	F: AGATCCAGAGCGCCTACAGCAA
	R: CAGGTCAGCATCAAGTCTTCGG
GAPDH	F: CATCACTGCCACCCAGAAGACTG
	R: ATGCCAGTGAGCTTCCCGTTCAG
U6	F: CTCGCTTCGGCAGCACAT
	R: TTTGCGTGTCATCCTTGCG

*RT-qPCR, reverse transcription-quantitative polymerase chain reaction; ETS-1, ETS proto-oncogene 1; HOXA13, homeobox A13; RUNX2, Runt-related gene 2; OCN, osteocalcin; OSX, osterix; GAPDH, glyceraldehyde-3-phosphate dehydrogenast.*

### Western Blot Analysis

Protein concentration was examined using a bicinchoninic acid kit (Thermo Fisher) after the protein extraction using the radio-immunoprecipitation assay (RIPA) cell lysis buffer (Solarbio Science and Technology Co., Ltd., Beijing, China). An equal volume of protein was separated on 12% SDS-PAGE and transferred on polyvinylidene fluoride membranes (Millipore Corp, Billerica, MA, United States). Then, the membranes were cultured with 5% non-fat milk and incubated with the primary antibodies at 4°C overnight, and then with the HRP-labeled secondary antibody at 20°C for 2 h. The protein signal intensity was detected using the enhanced chemiluminescence reagent (Pierce, Rockford, IL, United States). The primary antibodies were ETS1 (1:1,000, ab220361, Abcam), Runt-related gene 2 (RUNX2, 1:1,000, #12556, Cell Signaling Technology), osterix (OSX, 1:1,000, ab209484, Abcam), osteocalcin (OCN, 1:500, ab93876, Abcam), HOXA13 (1:1,000, ab172570, Abcam), β-catenin (1:5,000, ab32572, Abcam) and GAPDH (1:10,000, ab181602, Abcam), and the secondary antibody used was goat anti-rabbit IgG H&L (HRP) (1:10,000, ab205718, Abcam).

### Alkaline Phosphate (ALP) Staining

After 7 days of osteogenic differentiation, cells were immobilized in 4% paraformaldehyde for 10 min and stained using a BCIP/NBT Alp Color Development Kit (Beyotime, Shanghai, China) as per the kit’s protocols for 30 min without light exposure. Then, the images were captured using a digital camera, and the relative ALP expression was evaluated by the Image J software.

### Alizarin Red S (ARS) Staining

On day 21 following cultivation in osteogenic medium, the MC3T3-E1 cells were immobilized in ice-cold 70% ethanol at 4°C for 40 min and stained in 1% ARS (Sigma, St. Louis, MO, United States) at 20°C for 15 min. Next, the cells were washed with double-distilled water and imaged using the digital camera, and the size of bone mineralization was evaluated using the Image J software.

### Dual-Luciferase Reporter Gene Assay

The putative binding sequence between ETS1 and miR-128 promoter was first predicted on Jaspar^1^. Next, the promoter sequence of miR-128 was inserted into the pGL3-Basic luciferase reporter vector (Promega, Corp., Madison, WI, United States), and pGL3-control vector was used as the positive control. The vectors were then co-transfected with oe-NC or oe-ETS1 into 293T cells (purchased from ATCC). As for the binding relationship between miR-128 and HOXA13 3′UTR, the binding site between miR-128 and HOXA13 mRNA was first predicted on TargetScan. Then, the HOXA13 3′UTR (5′-GACAUUCAGCACACUGUGAAAAUGUAUUUGUGCACC UGCUUUUU-3′) containing the putative binding sequence with miR-128 was amplified and inserted into the pGL3-Promoter vectors (Promega) to construct HOXA13 wild type (WT) vectors, and the mutant type (MT) vectors containing the mutant binding sequences were constructed accordingly. Next, well-constructed WT and MT vectors were co-transfected with miR-128 mimic or NC mimic in 293T cells as well. For both binding relationships, cells were collected 48 h after transfection, and the relative luciferase activity in cells was determined using a Dual Luciferase Reporter Gene System (Promega).

### Chromatin Immunoprecipitation (ChIP) Assay

The binding relationship between ETS1 and miR-128 was further validated by an EZ-Magna ChIP kit (Millipore). Briefly, the MC3T3-E1 cells were crosslinked in 1% formaldehyde for 10 min and then quenched in glycine. Then, the DNA fragments were obtained following ultrasonic treatment. The A/G Agarose magnetic beads were conjugated with either anti-ETS1 (ab220361, Abcam) or anti-IgG (ab172730, Abcam), and then warm-incubated with the samples to allow the antibodies to bind to the targeting proteins. Then, the immunoprecipitated protein on the magnet beads was isolated and eluted, and the DNA enrichment in the elution was analyzed. The enrichment of miR-128 promoter was determined by qPCR. The corresponding primer sequence was (5′–3′): forward: GCTATTCCGCAACGT; reverse: CTCATGCTCCTAGCT (detection region: chr9:112119463–112119478).

### RNA Immunoprecipitation (RIP) Assay

A Magna RIP RNA-Binding Protein Immunoprecipitation kit (Millipore) was used for RIP assay in strict accordance with the kit’s protocols. In brief, after PBS washes, MC3T3-E1 cells were lysed in RIP lysis buffer and then centrifuged with the supernatant collected. The collected samples were incubated with anti-AGO2- (ab32381, Abcam) and anti-IgG (ab172730, Abcam)-coupled A/G agarose particles for RIP. The precipitated RNA was isolated using TRIzoL reagents, and the gene expression was determined by RT-qPCR.

### Statistical Analysis

Data were collected from three independent experiment and presented as mean ± standard deviation (SD). Differences between two groups were analyzed by unpaired *t*-test; while those among multiple groups were compared by one-way or two-way analysis of variance (ANOVA), followed by Tukey’s multiple comparison test. All data were analyzed using a GraphPad Prism 8 software (GraphPad Software, CA, United States). *p* < 0.05 was regarded to show statistically significant difference.

## Results

### ETS1 Is Upregulated During Osteogenic Differentiation

**FIGURE 1:**
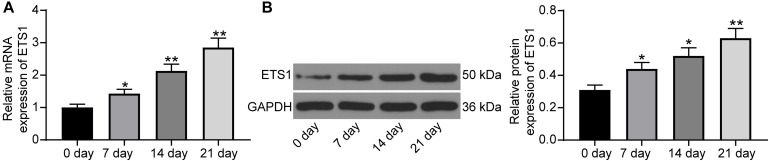
ETS1 is upregulated during osteogenic differentiation. **(A,B)** mRNA **(A)** and protein **(B)** expression of ETS1 in MC3T3-E1 cells during osteogenic differentiation measured by RT-qPCR and western blot analysis, respectively (one-way ANOVA, *p < 0.05; **p < 0.01, compared to day 0).

As previously discussed, ETS is a transcription factor crucial for bone formation and development, but its function on OP progression remains unexplained yet. Here, we first confirmed that the mRNA expression of ETS1 was gradually increased in MC3T3-E1 cells during osteogenic differentiation (Figure 1A), and a similar trend was presented with regard to its protein level (Figure 1B).

### Overexpression of ETS1 Triggers Osteogenic Differentiation of MC3T3-E1 Cells

**FIGURE 2:**
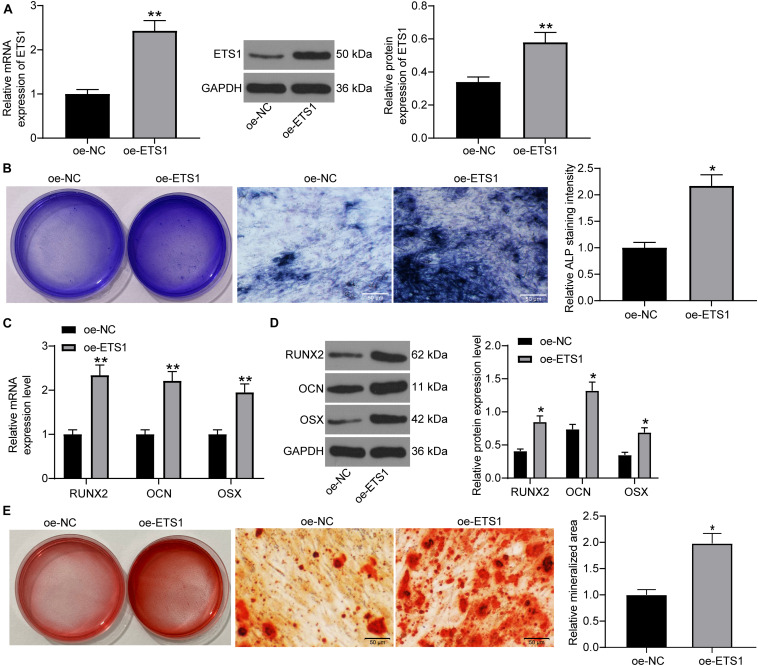
Overexpression of ETS1 promotes osteogenic differentiation of MC3T3-E1 cells. **(A)** Transfection efficacy of oe-ETS1 in MC3T3-E1 cells determined by RT-qPCR and western blot analysis (unpaired *t*-test, **p < 0.01). **(B)** After 7 days of osteogenic differentiation, the ALP expression in cells determined by ALP staining (osteogenic differentiation) (unpaired *t*-test, *p < 0.05). **(C,D)** mRNA **(C)** and protein **(D)** expression of OCN, OSX, and RUNX2 in cells determined by RT-qPCR and western blot analysis, respectively (two-way ANOVA, *p < 0.05; **p < 0.01). **(E)** ALP concentration in cells measured by ALP staining (unpaired *t*-test, *p < 0.05).

To further validate the impact of ETS1 on the osteogenic differentiation ability of MC3T3-E1 cells, overexpression of ETS1 was introduced in cells through the transfection of oe-ETS1, and the successful transfection was confirmed by the RT-qPCR and western blot results concerning ETS1 expression (Figure 2A). Transfected cells were cultured in medium for osteogenic differentiation induction. On day 7, the ALP staining results confirmed a significant increase in ALP expression in cells upon ETS1 overexpression (Figure 2B). In addition, we found that oe-ETS1 enhanced the levels of osteogenic markers including OCN, OSX and RUNX2 (Figures 2C,D). On day 21, we found an increased accumulation of calcareous in MC3T3-E1 cells by oe-ETS1 according to the ARS staining (Figure 2E). These results collectively suggest a promoting effect of ETS1 on osteogenic differentiation ability of MC3T3-E1 cells.

### ETS1 Transcriptionally Suppresses miR-128 Expression

**FIGURE 3:**
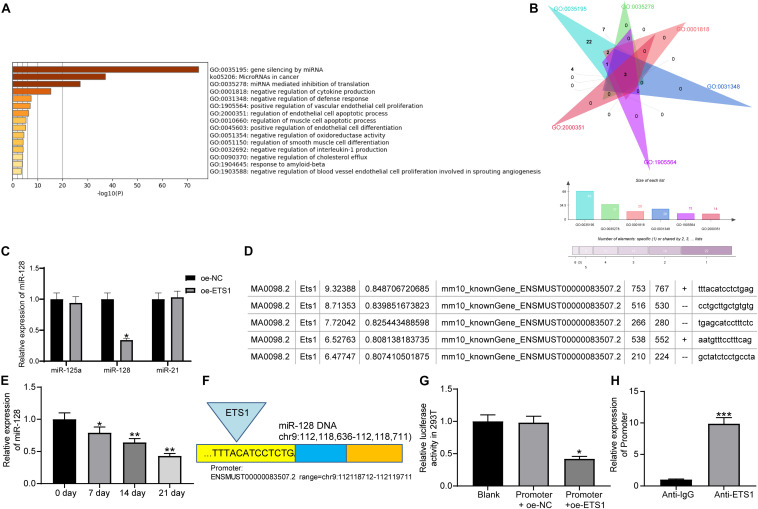
ETS1 transcriptionally suppresses miR-128 expression. **(A)** Candidate target miRNAs of ETS1 predicted on the hTFtarget system. **(B)** Common miRNAs enriched on the six most enriched signaling pathways according to the GO enrichment analysis. **(C)** Expression of miR-125a, miR-128 and miR-21 in MC3T3-E1cells overexpressing ETS1 examined by RT-qPCR (two-way ANOVA, *p < 0.05). **(D)** Putative binding sequence between ETS1 and miR-128 obtained from the Jaspar system. **(E)** MiR-128 expression in MC3T3-E1 cells during osteogenic differentiation measured by RT-qPCR (one-way ANOVA, *p < 0.05; **p < 0.01, compared to day 0). **(F)** The Promoter sequence of miR-128 selected for luciferase assay. **(G)** Binding relationship between ETS1 and miR-128 promoter determined by luciferase assay (one-way ANOVA, compared to Promoter + oe-NC, *p < 0.05). **(H)** Promoter enrichment in the precipitate compound pulled down by anti-ETS1 (unpaired *t*-test, ***p < 0.001).

As aforementioned, ETS1 can mediate the promoter region of miRNAs to govern their expression (Harris et al., 2010; Kern et al., 2012). But such mediation network has hardly been investigated in the osteogenesis process. We first predicted the potential target miRNAs of ETS1 on the hTFtarget system^2^, and performed pathway enrichment analyses based on the miRNAs on the Metascape system^3^ (Figure 3A). The top six enriched signaling pathway, suggested by Gene Ontology (GO) enrichment analysis, were focused and further analyzed. Three common miRNAs including miR-125a, miR-128, and miR-21 were found to be enriched on these signaling pathways (Figure 3B). Then, we examined the expression of miR-125a, miR-128 and miR-21 in MC3T3-E1 cells overexpressing ETS1. It was found that overexpression of ETS1 significantly reduced the expression of miR-128 while it had little effect on the expression of miR-125a or miR21 (Figure 3C).

Data on the Jaspar System suggested possible binding sites between ETS1 and the promoter region of miR-128 (Figure 3D), while miR-128 has once been reported to possibly promote OP progression (Zhao et al., 2019). We therefore speculated that ETS1 may exert promoting functions through regulating miR-128.

We next measured the expression of miR-128 during osteogenic differentiation of MC3T3-E1 cells. The RT-qPCR results showed that the miR-128 expression was gradually reduced in this process (Figure 3E).

To validate the binding relationship between ETS1 and the miR-128 promoter, we selected the predicted promoter sequence with highest score on the Jasper Database, termed Promoter (Figure 3F). The Promoter was inserted into the luciferase reporter vector and co-transfected with oe-ETS1 or oe-NC into 293T cells. It was found that co-transfection of oe-ETS1 reduced the luciferase activity of the Promoter (Figure 3G). In addition, a ChIP assay was further carried out, which showed that abundant promoter was enriched by anti-ETS1 in the precipitates compared to that by anti-IgG (Figure 3H), further indicating that ETS1 can bind to the promoter of miR-128.

### Overexpression of miR-128 Blocks the Promotion of ETS1 on Osteogenic Differentiation

**FIGURE 4:**
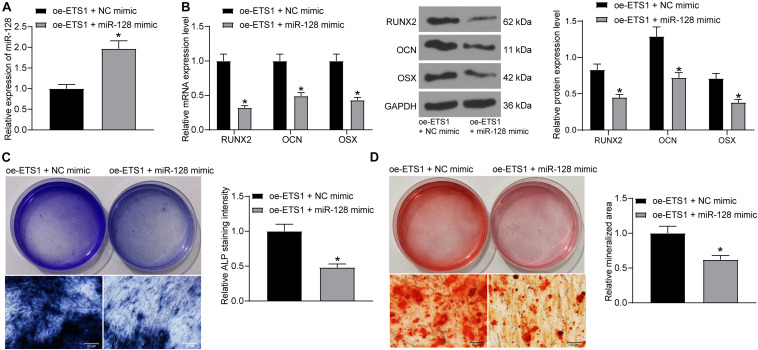
Overexpression of miR-128 blocks the promotion of ETS1 on osteogenic differentiation. **(A)** Transfection efficacy of miR-128 mimic determined by RT-qPCR (unpaired *t*-test, *p < 0.05). **(B)** mRNA and protein levels of osteogenic markers including OCN, OSX and RUNX2 in cells determined by RT-qPCR and western blot analysis, respectively (two-way ANOVA, *p < 0.05). **(C)** ALP concentration in MC3T3-E1 cells measured by ALP staining (unpaired *t*-test, *p < 0.05). **(D)** Calcareous accumulation in MC3T3-E1 cells measured by ARS staining (unpaired *t*-test, *p < 0.05).

To further explore the effects of miR-128 expression on osteogenic differentiation, overexpression of miR-128 was further introduced in MC3T3-E1 cells overexpressing ETS1, and the transfection efficacy was validated by RT-qPCR again (Figure 4A). Next, the cells were incubated for osteogenic differentiation. On day 7, the mRNA and protein levels of RUNX2, OCN, and OSX in cells were notably decreased upon miR-128 overexpression (Figure 4B). In addition, the ALP staining results showed that overexpression of miR-128 led to a significant decline in ALP concentration in cells (Figure 4C). On day 21, the ARS staining results showed that the increased accumulation of calcareous in MC3T3-E1 cells by oe-ETS1 was reduced by further administration of miR-128 mimic (Figure 4D).

### miR-128 Directly Binds to HOXA13

**FIGURE 5:**
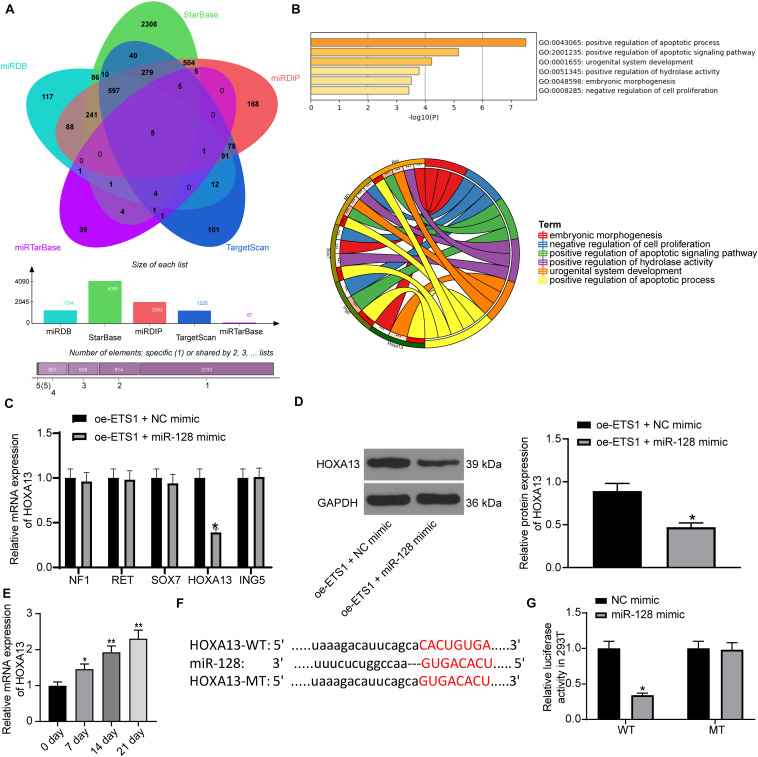
miR-128 directly binds to HOXA13. **(A)** Candidate target transcripts of miR-128 predicted using five bioinformatic systems. **(B)** A GO pathway enrichment analysis based on the predicted genes. **(C)** mRNA expression of NF1, RET, SOX7, HOXA13, and ING5 in cells transfected with oe-ETS1 + NC mimic examined by RT-qPCR (two-way ANOVA, *p < 0.05). **(D)** Protein level of HOXA13 in cells transfected with oe-ETS1 + NC mimic determined by western blot analysis (unpaired *t*-test, *p < 0.05). **(E)** mRNA expression of HOXA13 in MC3T3-E1 cells during osteogenic differentiation examined using RT-qPCR (one-way ANOVA, *p < 0.05, **p < 0.01). **(F)** Binding site between miR-128 and HOXA13 predicted on TargetScan. **(G)** Binding relationship between miR-128 and HOXA13 mRNA validated by a luciferase assay (two-way ANOVA, *p < 0.05).

Following the findings above, we further predicted the target mRNAs of miR-128 using five bioinformatic systems including miRDB^4^, StarBase^5^, miRDIP^6^, TargetScan^7^ and miRTarBase^8^, and five common candidate targets were predicted: NF1, RET, SOX7, HOXA13, and ING5 (Figure 5A). The subsequent pathway enrichment analysis suggested that these genes were all enriched on the GO:0043065 signaling pathway (Figure 5B). We then examined the mRNA expression of these genes in cells transfected with oe-ETS1 + miR-128 mimic using RT-qPCR (Figure 5C). It was found that miR-128 mimic significantly reduced the expression of HOXA13 mRNA while it had little effect on the expression of the other four transcripts. Likewise, the western blot analysis results showed that overexpression of miR-128a reduced the protein level of HOXA13 (Figure 5D). Then, the RT-qPCR results suggested that the expression of HOXA13 was gradually increased during the process of osteogenic differentiation of MC3T3-E1 cells (Figure 5E).

Thereafter, we obtained the putative binding site between HOXA13 and miR-128 from TargetScan, and the mutant type (MT) sequence was designed as well (Figure 5F) for luciferase assays. The HOXA13-WT and HOXA13-MT luciferase vectors were co-transfected with miR-128 mimic or NC mimic into 293T cells. It was found that miR-128 reduced the luciferase activity of HOXA13-WT vector in cells, while it had no significant effect on luciferase activity of HOXA13-MT vector in cells (Figure 5G).

### HOXA13 Promotes Osteogenic Differentiation Ability of MC3T3-E1 Cells

**FIGURE 6:**
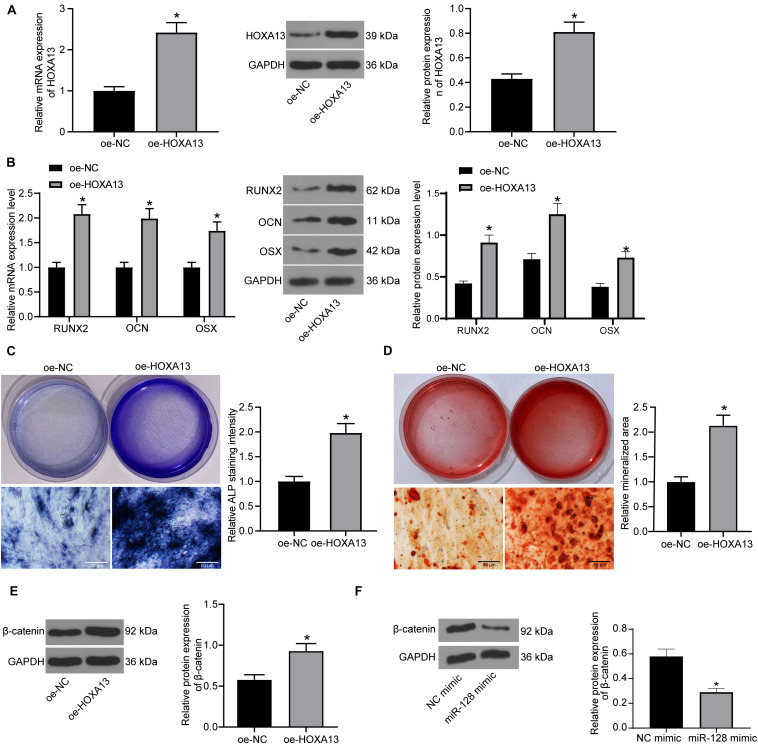
HOXA13 promotes osteogenic differentiation ability of MC3T3-E1 cells. **(A)** Transfection efficacy of oe-HOXA13 determined by RT-qPCR and western blot analysis (unpaired *t*-test, *p < 0.05). **(B)** mRNA and protein expression of RUNX2, OCN and OSX in cells on day 7 determined by RT-qPCR and western blot analysis, respectively (two-way ANOVA, *p < 0.05). **(C)** ALP concentration in cells measured by ALP staining (unpaired *t*-test, *p < 0.05). **(D)** Calcareous accumulation in cells on day 21 evaluated by ARS staining (unpaired *t*-test, *p < 0.05). **(E,F)** Protein level of β-catenin in cells after HOXA13 **(E)** or miR-128 **(F)** overexpression determined by western blot analysis (unpaired *t*-test, *p < 0.05).

Based on the above findings, we further transfected oe-HOXA13 in MC3T3-E1 cells, and the RT-qPCR and western blot analysis results showed that the transfection was successfully performed (Figure 6A). Then, the cells were cultured for osteogenic differentiation. It was found that the expression of RUNX2, OCN, and OSX in cells was increased on day 7 upon HOXA13 upregulation (Figure 6B). Still, the ALP concentration in cells (Figure 6C), and the calcareous accumulation in MC3T3-E1 cells on day 21 (Figure 6D) were increased after oe-HOXA13 transfection.

Intriguingly, HOXA13 was reported as a positive regulator of β-catenin (Qu et al., 2017), which is a well-recognized signaling pathway involved in multiple aspects of growth and development in many organs including bone and tooth formation (Duan and Bonewald, 2016). We therefore speculated that HOXA13 promotes osteogenic differentiation with the possible implication of the Wnt/β-catenin signaling pathway. Hence, we explored the protein expression of β-catenin in cells after miR-128 mimic or oe-HOXA13 transfection using western blot analysis. It was found that the protein level of β-catenin was significantly reduced after miR-128 upregulation but enhanced after miR-128 inhibition (Figures 6E,F).

### Inhibition of β-Catenin Blocks the Promotive Effects of oe-ETS1 on Osteogenic Differentiation

**FIGURE 7:**
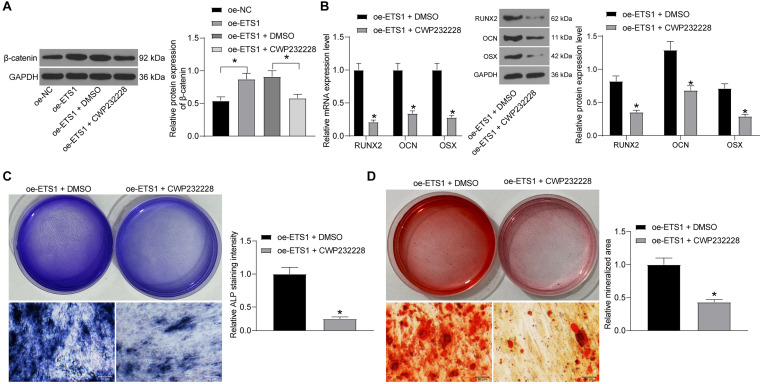
Inhibition of β-catenin blocks the promotion of oe-ETS1 on osteogenic differentiation. **(A)** Protein expression of β-catenin in MC3T3-E1 cells after CWP232228 administration determined by western blot analysis (one-way ANOVA, *p < 0.05). **(B)** mRNA and protein expression of RUNX2, OCN, and OSX in cells on day 7 determined by RT-qPCR and western blot analysis, respectively (two-way ANOVA, *p < 0.05). **(C)** ALP concentration in cells measured by ALP staining (unpaired *t*-test, *p < 0.05). **(D)** Calcareous accumulation in cells on day 21 evaluated by ARS staining (unpaired *t*-test, *p < 0.05).

To confirm whether β-catenin is involved in the above events, we further administrated CWP232228, a β-catenin-specific antagonist, into MC3T3-E1 cells overexpressing ETS1, and cells transfected with DMSO were set as control. It was found that the protein level of β-catenin was notably decreased by CWP232228 (Figure 7A). Next, the cells with stable transfection were cultured for osteogenic differentiation. On day 7, the levels of RUNX2, OCN, and OSX in cells were notably declined by CWP232228 (Figure 7B). Likewise, the ALP concentration in cells on day 7 (Figure 7C), and the calcareous accumulation in cells on day 21 (Figure 7D) were notably reduced upon β-catenin downregulation. These results indicated that the Wnt/β-catenin signaling pathway is, at least partially, involved in the osteogenic differentiation promotion by ETS1.

## Discussion

OP has no typical clinical presentation until there is a fracture, and it is estimated that more than 90 million people in China suffer from OP and this number will rise to 221 million by 2050 (Shi and Zhang, 2019). This chronic disease largely reduces the life quality and expectance of individuals, and burdens heavily to the health care systems, especially there is no current effective treatment to cure the bone loss. In the present study, we identified that ETS1 suppresses miR-128 transcription and subsequently upregulates HOXA13 expression, which promotes the osteogenic differentiation ability of MC3T3-E1 cells with the presence of increased ALP expression and mineralization, and the elevated expression of osteogenic biomarkers including OCN, OSX, and RUNX2.

The versatile functions of ETS1 on osteogenesis have been demonstrated. ETS1 was abundantly expressed in proliferating pre-osteoblastic cells and responsible for retinoic acid-mediated skeletal growth and development as well as bone turnover (Raouf and Seth, 2000). The initial finding of this study was that the mRNA and protein expression of ETS1 was increased during the process of osteogenic differentiation of MC3T3-E1 cells. A previous study noted that ETS1 transcriptionally regulates RING finger protein RNF11, which was increased during three major stages including proliferation, differentiation, and mineralization (Gao et al., 2005). As aforementioned, OP is attributed to the loss of bone mass due to the imbalance between osteoblast-mediated bone formation and osteoclast-controlled bone absorption, and the former one presents various characteristics during differentiation such as increased ALP activity and the following extracellular matrix synthesis and mineralization (Zhou et al., 2020). Here, our study further noted that overexpression of ETS1 led to an increase in ALP expression and the mineralization of MC3T3-E1 cells, as well as the expression of RUNX2, a central factor regulating osteoblast differentiation and triggering bone mineralization (Hou et al., 2019), and some other osteogenic biomarkers including OCN and OSX (Yi et al., 2019). Collectively, these results validated a promoting role of ETS1 in the osteogenic differentiation of MC3T3-E1 cells.

miRNAs are increasingly recognized as key molecules involved in bone remodeling or OP development (Feng et al., 2018). As a transcript, ETS1 itself can be regulated by miRNA and its inhibition was found to suppress osteogenic differentiation in MC3T3-E1 cells (Itoh et al., 2010; Fan et al., 2020). In addition to this, as a transcription factor, it can mediate the transcription of downstream transcripts including miRNAs (Taylor et al., 2016), though such interaction has not been reported in osteogenic differentiation yet. Here, the data on the Jaspar system suggested ETS1 had a potential binding relationship with the promoter region of miR-128. Then, the integrated expression detection, luciferase assay, ChIP assay validated this binding relationship. miR-128 has been reported to promote OP development (Zhao et al., 2019). Likewise, miR-128-3p was found to be responsible for mesenchymal stem cell-derived exosomes-mediated mitigation in osteogenic differentiation and fracture healing (Xu et al., 2020). More recently, a study by Shen et al. (2020) reported a crucial function of miR-128 in murine osteoclastogenesis and estrogen deficiency-induced bone loss. Importantly, here in our study, we found that overexpression of miR-128 reversed the promotion of ETS1 on the osteogenic differentiation of MC3T3-E1 cells, indicating that downregulation of miR-128 was, at least partially, responsible for ETS1-mediated osteogenesis.

Next, according to the data predicted on TargetScan and the following expression detection and luciferase assay, we found that miR-128 could directly bind to HOXA13. Although there is limited information concerning the correlation between HOXA13 and osteogenic differentiation or OP development, studies have noted the important function of one of its homologous genes, HOXA10, in the promotion of osteogenesis in bone marrow stem cells (Liao et al., 2013) and in the alleviation in unloading-induced bone loss (Wang et al., 2020). Here, we observed that overexpression in MC3T3-E1 cells increased the expression of ALP, RUNX2, OCN, and OSX as well as the mineralization. In addition, HOXA13 was reported as an upstream regulator of β-catenin (Qu et al., 2017), which has been reported as a predominating regulator of osteogenesis as well as bone morphogenetic proteins, and therefore a promising intervention for OP control (Xia et al., 2020). This potent regulation of Wnt/β-catenin in osteogenesis has also been increasingly evidenced in some recent reports (Molagoda et al., 2019; Zhang et al., 2019; Xia et al., 2020). To validate whether this pathway is implicated in the above events mediated by the ETS1/miR-125/HOXA13 axis, we first determined the correlation between HOXA13 expression and the β-catenin activity in cells. Overexpression of HOXA13 activated the Wnt/β-catenin pathway. Importantly, the involvement of Wnt/β-catenin was further confirmed by a rescue experiment where administration of CWP232228 blocked the promoting roles of ETS1 in osteogenesis.

## Conclusion

**FIGURE 8:**
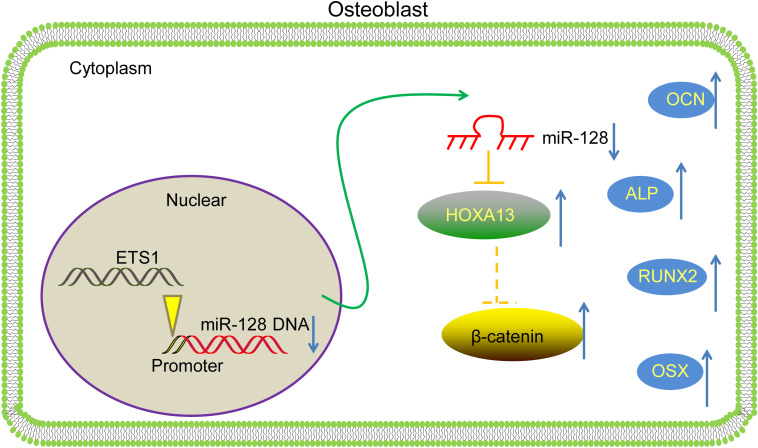
A diagram for molecular mechanism. In MC3T3-E1 cells, ETS1 specifically binds to the promoter region of miR-128 to suppress its transcription, leading to further activation of the HOXA13/β-catenin axis, therefore promoting the osteogenic differentiation of cells.

To conclude, this study evidenced that ETS1 could transcriptionally suppress miR-128 to upregulate HOXA13 expression, which promotes osteogenic differentiation and mineralization in MC3T3-E1 cells with the involvement of Wnt/β-catenin activation (Figure 8). We hope this study can offer novel insights into OP control.

## Data Availability Statement

The original contributions presented in the study are included in the article/supplementary material, further inquiries can be directed to the corresponding author/s.

## Author Contributions

RL was the guarantor of integrity of the entire study and contributed to the concepts and contributed to the manuscript preparation. YD and FL contributed to the experimental studies, data acquisition, and statistical analysis. All authors contributed to the article and approved the submitted version.

## Conflict of Interest

FL was employed by company Naton Biotech (Beijing) Co. The remaining authors declare that the research was conducted in the absence of any commercial or financial relationships that could be construed as a potential conflict of interest.

## Footnotes

1 http://jaspar.genereg.net

2 http://bioinfo.life.hust.edu.cn/hTFtarget/#!/

3 https://metascape.org/

4 http://mirdb.org/

5 http://starbase.sysu.edu.cn/

6 http://ophid.utoronto.ca/mirDIP/

7 http://www.targetscan.org/vert_72/

8 http://mirtarbase.cuhk.edu.cn/php/search.php
